# Cardiac Effects of Trimetazidine in Diabetic Rats

**DOI:** 10.5935/abc.20190012

**Published:** 2019-02

**Authors:** Alfredo J. Mansur

**Affiliations:** Instituto do Coração (InCor), Hospital das Clínicas da Faculdade de Medicina da Universidade de São Paulo, São Paulo, SP - Brazil

**Keywords:** Diabetes Mellitus, Diabetic Cardiomyopathies, Hypertrophic, Left Ventricular/metabolismo, Oxidative Stress, Angina Pectoris/metabolismo, Trimetazidine/therapeutic use

Diabetes mellitus may be associated with a specific form of cardiomyopathy independent of other comorbidities. Left ventricular hypertrophy is the main pathological change described in cardiomyopathy of patients with diabetes mellitus. Different mechanisms may be operative in the pathogenesis of the hypertrophy: metabolic derangements, inflammation and among other factors, oxidative stress.^1^ Oxidative stress may lead to cellular damage by free radical-induced oxidation of DNA, proteins and lipids.^2^

Reactive oxygen species may lead to consequences in cardiomyocytes including hypertrophy, apoptosis and fibrosis; in this regard a cellular detoxification mechanism may be missing for attenuating severity of damage induced by some reactive oxygen species.^3^ Hence, interventions that might be protective would be worth to be investigated, including drug therapy.

Trimetazidine (1-[2,3,4-trimethoxybenzyl] piperazine dihydrochloride)^4^ is one of the drugs that may be used in combination with other drugs for the treatment of patients with angina pectoris.^5^ It is a piperazine derivative^6^ characterized as a metabolic modulator^5^ that reduces long-chain fatty acid (3-ketoactyl CoA thiolase) oxidation.^4^

In this issue,^7^ an experimental study of alloxan induced diabetes in Sprague-Dawley rats tested the hypothesis that the administration of trimetazidine might prevent pathologic changes induced in the heart of the studied animals, including QT interval, heart weight relative to body weight, myocardial contractility indices and antioxidant enzyme activities (superoxide dismutase, catalase and glutathione peroxidase). The authors found that the administration of trimetazidine reduced the modifications in the studied variables induced by diabetes in the rats. Thus, additional experimental findings were added to current knowledge about the interaction between diabetes, cardiomyopathy and drug therapy in experimental animals, rats in this specific study. In the event of progress in accumulating knowledge together with other studies, evidences may evolve to deserving clinical studies.
